# Experimental fatigue dataset for additive-manufactured 3D-printed Polylactic acid biomaterials under fully-reversed rotating-bending bending loadings

**DOI:** 10.1016/j.dib.2022.107846

**Published:** 2022-01-21

**Authors:** Mohammad Azadi, Ali Dadashi

**Affiliations:** Faculty of Mechanical Engineering, Semnan University, Semnan, Iran

**Keywords:** Fatigue dataset, Additive manufacturing, 3D-printing, Polylactic acid biomaterial, Cyclic loading

## Abstract

In this dataset, experimental fatigue testing results have been presented for the additive-manufactured 3D-printed Polylactic acid (PLA) biomaterials under fully-reversed rotating-bending loadings. For such an objective, a fused deposition modeling (FDM) 3D-printer was utilized to fabricate the standard cylindrical samples with different printing parameters in the horizontal direction. For the demonstration of printing parameter effects on the PLA fatigue lifetime, the nozzle diameters were from 0.2 to 0.6 mm, the extruder temperatures were from 180 to 240 °C, and finally, the printing speeds were from 5 to 15 mm/s. Then after 3D-printing of specimens, fatigue testing was performed on various samples under fully-reversed rotating-bending loadings. Then, the fatigue data were presented in tables through the high-cycle fatigue regime, under the load-controlled condition. For further works, these dataset tables could be used to draw the S-N (stress-lifetime) diagram, to find the fatigue strength coefficient and exponent.

## Specifications Table

**Table:** 

Subject	Engineering
Specific subject area	Engineering/ Bioengineering/ Manufacturing Engineering/ Mechanical Engineering/ Automotive Engineering/Fatigue of polymers/ Additive Manufacturing
Type of data	Table
How the data were acquired	The data were acquired by the rotary bending fatigue testing device, including the fatigue lifetimes of standard samples, which were 3D-printed under various process parameters. PLA specimens were firstly fabricated by additive manufacturing in horizontal direction and then, were exposed to fully-reversed rotating-bending loadings until the fracture. The number of cycles to failure was accounted for and reported in tables for each sample, under different stress levels.
Data format	Raw
Description of data collection	For the fused deposition modeling (FDM) 3D-printing, 3 process parameters were considered as follows, the nozzle diameter: 0.2, 0.4, and 0.6 mm, the printing temperature: 180, 210, and 240 °C, and the printing speed: 5, 10, and 15 mm/s. After the fabrication of standard samples, fatigue testing was done under fully-reversed rotating-bending loadings, under different stress levels, from 2.5 to 17.5 MPa.
Data source location	• Institution: Faculty of Mechanical Engineering, Semnan University • City/Town/Region: Semnan • Country: Iran • Latitude and longitude (and GPS coordinates, if possible) for collected samples/data: 35.59878671018807, 53.433229370400255
Data accessibility	All data referred to in this document are available in tables.

## Value of the Data

• Since the main problem in additive-manufactured components is the quality; therefore, various parameters of the 3D-printing process should be evaluated by engineers. Having a dataset on the fatigue lifetime for such parts, could be helpful and also useful.
• The benefit of these data is to find the effect of different parameters in the 3D-printing technique on the quality in general and on the fatigue lifetime and strength (as a specific criterion) of PLA biomaterials. This experimental fatigue data could help design engineers to further investigations, based on a known behavior of the material.
• Using these fatigue datasets could increase the knowledge of the PLA biomaterial behavior, fabricated by fused deposition modeling (FDM) 3D-printers. The data could be utilized to demonstrate the S-N (stress-lifetime) of the material under different fabrication variables in order to find the superior condition of 3D-printing.
• The main concern in this dataset is the fatigue strength and lifetime of PLA biomaterials. Such data could be rarely found in articles and this could be one novelty of the presented experimental results. Several articles were presented on the tensile properties of PLA; however, fatigue properties are still rare, especially under various parameters of the 3D-printing process.
• These fatigue datasets could be reused for further developments of experiments, as the initial analysis of the fatigue lifetimes for PLA biomaterials. In the future, the fatigue strength of the novel materials could be compared to these data. The novel material could be introduced by reinforcing PLA with fibers or other improvement techniques for the material strength and lifetime.

## 1 Data Description

Raw data for additive-manufactured PLA biomaterials could be seen in Table 1, Table 2, Table 3, Table 4, Table 5, Table 6, Table 7, Table 8, Table 9, Table 10, Table 11, Table 12, Table 13, Table 14, Table 15, Table 16, Table 17, Table 18, Table 19, Table 20, Table 21, Table 22, Table 23, Table 24, Table 25, Table 26, Table 27. These experimental fatigue lifetimes under different 3D-printing parameters were presented without any pre-analysis and filtering. Only as an initial filtration, some descriptions are added in the last column, including “RO” as the “Run-out” and “OSB” as the “Out of Scatter-band”. The “Run-out” one means the fatigue lifetime was more than 1.5 million cycles and fatigue testing did not continue. Notably, other fatigue experiments continued until the sample was fractured. The “Out of Scatter-band” data, only as a preliminary analysis, had high scattering, which may be due to the poor quality of the 3D-printed specimen.

**Table 1:** Fatigue testing results for 3D-printed samples with a printing speed of 5 mm/s at 180 °C and a nozzle diameter of 0.2 mm.

No.	Material	Diameter	Speed	Temperature	Stress	Lifetime	Description
[-]	[-]	[mm]	[mm/s]	[˚C]	[MPa]	[cycle]	[-]
1	PLA-0.2-5-180	0.2	5	180	5.0	942000	
2	PLA-0.2-5-180	0.2	5	180	7.5	600	OSB
3	PLA-0.2-5-180	0.2	5	180	7.5	597800	
4	PLA-0.2-5-180	0.2	5	180	10.0	400	OSB
5	PLA-0.2-5-180	0.2	5	180	10.0	148900	
6	PLA-0.2-5-180	0.2	5	180	12.5	1000	OSB

**Table 2:** Fatigue testing results for 3D-printed samples with a printing speed of 5 mm/s at 210 °C and a nozzle diameter of 0.2 mm.

No.	Material	Diameter	Speed	Temperature	Stress	Lifetime	Description
[-]	[-]	[mm]	[mm/s]	[˚C]	[MPa]	[cycle]	[-]
1	PLA-0.2-5-210	0.2	5	210	2.5	1,500,000	RO
2	PLA-0.2-5-210	0.2	5	210	5.0	1,500,000	RO
3	PLA-0.2-5-210	0.2	5	210	5.0	1,500,000	RO
4	PLA-0.2-5-210	0.2	5	210	5.0	500	OSB
5	PLA-0.2-5-210	0.2	5	210	7.5	3000	OSB
6	PLA-0.2-5-210	0.2	5	210	7.5	78,200	
7	PLA-0.2-5-210	0.2	5	210	7.5	69,500	
8	PLA-0.2-5-210	0.2	5	210	10.0	61,900	
9	PLA-0.2-5-210	0.2	5	210	10.0	41,500

**Table 3:** Fatigue testing results for 3D-printed samples with a printing speed of 5 mm/s at 240 °C and a nozzle diameter of 0.2 mm.

No.	Material	Diameter	Speed	Temperature	Stress	Lifetime	Description
[-]	[-]	[mm]	[mm/s]	[˚C]	[MPa]	[cycle]	[-]
1	PLA-0.2-5-240	0.2	5	240	2.5	1,500,000	RO
2	PLA-0.2-5-240	0.2	5	240	5.0	1,300	OSB
3	PLA-0.2-5-240	0.2	5	240	5.0	1,500,000	RO
4	PLA-0.2-5-240	0.2	5	240	5.0	714,000	
5	PLA-0.2-5-240	0.2	5	240	7.5	500	OSB
6	PLA-0.2-5-240	0.2	5	240	7.5	500	OSB
7	PLA-0.2-5-240	0.2	5	240	7.5	278,800	
8	PLA-0.2-5-240	0.2	5	240	7.5	176,300	
9	PLA-0.2-5-240	0.2	5	240	10.0	600	OSB
10	PLA-0.2-5-240	0.2	5	240	10.0	98,000

**Table 4:** Fatigue testing results for 3D-printed samples with a printing speed of 10 mm/s at 180 °C and a nozzle diameter of 0.2 mm.

No.	Material	Diameter	Speed	Temperature	Stress	Lifetime	Description
[-]	[-]	[mm]	[mm/s]	[˚C]	[MPa]	[cycle]	[-]
1	PLA-0.2-10-180	0.2	10	180	7.5	277,200	
2	PLA-0.2-10-180	0.2	10	180	10.0	96,300	
3	PLA-0.2-10-180	0.2	10	180	10.0	72,100	
4	PLA-0.2-10-180	0.2	10	180	12.5	400	OSB
5	PLA-0.2-10-180	0.2	10	180	12.5	45,900	
6	PLA-0.2-10-180	0.2	10	180	15.0	400	OSB

**Table 5:** Fatigue testing results for 3D-printed samples with a printing speed of 10 mm/s at 210 °C and a nozzle diameter of 0.2 mm.

No.	Material	Diameter	Speed	Temperature	Stress	Lifetime	Description
[-]	[-]	[mm]	[mm/s]	[˚C]	[MPa]	[cycle]	[-]
1	PLA-0.2-10-210	0.2	10	210	7.5	212,600	
2	PLA-0.2-10-210	0.2	10	210	10.0	600	OSB
3	PLA-0.2-10-210	0.2	10	210	10.0	177,000	
4	PLA-0.2-10-210	0.2	10	210	12.5	20,400	
5	PLA-0.2-10-210	0.2	10	210	12.5	400	OSB
6	PLA-0.2-10-210	0.2	10	210	15.0	18,000

**Table 6:** Fatigue testing results for 3D-printed samples with a printing speed of 10 mm/s at 240 °C and a nozzle diameter of 0.2 mm.

No.	Material	Diameter	Speed	Temperature	Stress	Lifetime	Description
[-]	[-]	[mm]	[mm/s]	[˚C]	[MPa]	[cycle]	[-]
1	PLA-0.2-10-240	0.2	10	240	5.0	1,500,000	RO
2	PLA-0.2-10-240	0.2	10	240	7.5	700	OSB
3	PLA-0.2-10-240	0.2	10	240	7.5	73,100	
4	PLA-0.2-10-240	0.2	10	240	7.5	121,000	
5	PLA-0.2-10-240	0.2	10	240	10.0	67,000	
6	PLA-0.2-10-240	0.2	10	240	12.5	21,500	
7	PLA-0.2-10-240	0.2	10	240	15.0	2,000	OSB

**Table 7:** Fatigue testing results for 3D-printed samples with a printing speed of 15 mm/s at 180 °C and a nozzle diameter of 0.2 mm.

No.	Material	Diameter	Speed	Temperature	Stress	Lifetime	Description
[-]	[-]	[mm]	[mm/s]	[˚C]	[MPa]	[cycle]	[-]
1	PLA-0.2-15-180	0.2	15	180	5.0	42,000	
2	PLA-0.2-15-180	0.2	15	180	7.5	13,000	
3	PLA-0.2-15-180	0.2	15	180	7.5	1,000	OSB
4	PLA-0.2-15-180	0.2	15	180	10.0	4,000	
5	PLA-0.2-15-180	0.2	15	180	10.0	500	OSB
6	PLA-0.2-15-180	0.2	15	180	12.5	3,000

**Table 8:** Fatigue testing results for 3D-printed samples with a printing speed of 15 mm/s at 210 °C and a nozzle diameter of 0.2 mm.

No.	Material	Diameter	Speed	Temperature	Stress	Lifetime	Description
[-]	[-]	[mm]	[mm/s]	[˚C]	[MPa]	[cycle]	[-]
1	PLA-0.2-15-210	0.2	15	210	5.0	2,000	OSB
2	PLA-0.2-15-210	0.2	15	210	5.0	1,500,000	RO
3	PLA-0.2-15-210	0.2	15	210	7.5	2,700	OSB
4	PLA-0.2-15-210	0.2	15	210	7.5	66,700	
5	PLA-0.2-15-210	0.2	15	210	7.5	1,000	OSB
6	PLA-0.2-15-210	0.2	15	210	10.0	146,500	
7	PLA-0.2-15-210	0.2	15	210	12.5	500	OSB
8	PLA-0.2-15-210	0.2	15	210	12.5	3,000

**Table 9:** Fatigue testing results for 3D-printed samples with a printing speed of 15 mm/s at 240 °C and a nozzle diameter of 0.2 mm.

No.	Material	Diameter	Speed	Temperature	Stress	Lifetime	Description
[-]	[-]	[mm]	[mm/s]	[˚C]	[MPa]	[cycle]	[-]
1	PLA-0.2-15-240	0.2	15	240	5.0	868,900	
2	PLA-0.2-15-240	0.2	15	240	7.5	41,000	
3	PLA-0.2-15-240	0.2	15	240	10.0	24,000	
4	PLA-0.2-15-240	0.2	15	240	12.5	1,400	
5	PLA-0.2-15-240	0.2	15	240	12.5	700	OSB
6	PLA-0.2-15-240	0.2	15	240	12.5	600	OSB

**Table 10:** Fatigue testing results for 3D-printed samples with a printing speed of 5 mm/s at 180 °C and a nozzle diameter of 0.4 mm.

No.	Material	Diameter	Speed	Temperature	Stress	Lifetime	Description
[-]	[-]	[mm]	[mm/s]	[˚C]	[MPa]	[cycle]	[-]
1	PLA-0.4-5-180	0.4	5	180	5.0	45,500	
2	PLA-0.4-5-180	0.4	5	180	7.5	21,400	
3	PLA-0.4-5-180	0.4	5	180	10.0	3,000	OSB
4	PLA-0.4-5-180	0.4	5	180	10.0	10,200	
5	PLA-0.4-5-180	0.4	5	180	12.5	6,000	
6	PLA-0.4-5-180	0.4	5	180	15.0	3,100

**Table 11:** Fatigue testing results for 3D-printed samples with a printing speed of 5 mm/s at 210 °C and a nozzle diameter of 0.4 mm.

No.	Material	Diameter	Speed	Temperature	Stress	Lifetime	Description
[-]	[-]	[mm]	[mm/s]	[˚C]	[MPa]	[cycle]	[-]
1	PLA-0.4-5-210	0.4	5	210	2.5	86,700	
2	PLA-0.4-5-210	0.4	5	210	5.0	75,000	
3	PLA-0.4-5-210	0.4	5	210	5.0	24,000	
4	PLA-0.4-5-210	0.4	5	210	7.5	2,200	
5	PLA-0.4-5-210	0.4	5	210	7.5	4,000	
6	PLA-0.4-5-210	0.4	5	210	10.0	1,600

**Table 12:** Fatigue testing results for 3D-printed samples with a printing speed of 5 mm/s at 240 °C and a nozzle diameter of 0.4 mm.

No.	Material	Diameter	Speed	Temperature	Stress	Lifetime	Description
[-]	[-]	[mm]	[mm/s]	[˚C]	[MPa]	[cycle]	[-]
1	PLA-0.4-5-240	0.4	5	240	2.5	500	OSB
2	PLA-0.4-5-240	0.4	5	240	2.5	2,200	
3	PLA-0.4-5-240	0.4	5	240	5.0	2,200	
4	PLA-0.4-5-240	0.4	5	240	5.0	2,700	
5	PLA-0.4-5-240	0.4	5	240	7.5	1,800	
6	PLA-0.4-5-240	0.4	5	240	7.5	1,700

**Table 13:** Fatigue testing results for 3D-printed samples with a printing speed of 10 mm/s at 180 °C and a nozzle diameter of 0.4 mm.

No.	Material	Diameter	Speed	Temperature	Stress	Lifetime	Description
[-]	[-]	[mm]	[mm/s]	[˚C]	[MPa]	[cycle]	[-]
1	PLA-0.4-10-180	0.4	10	180	5.0	108,000	
2	PLA-0.4-10-180	0.4	10	180	7.5	28,600	
3	PLA-0.4-10-180	0.4	10	180	7.5	34,400	
4	PLA-0.4-10-180	0.4	10	180	10.0	4,500	OSB
5	PLA-0.4-10-180	0.4	10	180	10.0	10,000	
6	PLA-0.4-10-180	0.4	10	180	12.5	12,800

**Table 14:** Fatigue testing results for 3D-printed samples with a printing speed of 10 mm/s at 210 °C and a nozzle diameter of 0.4 mm.

No.	Material	Diameter	Speed	Temperature	Stress	Lifetime	Description
[-]	[-]	[mm]	[mm/s]	[˚C]	[MPa]	[cycle]	[-]
1	PLA-0.4-10-210	0.4	10	210	5.0	13,100	OSB
2	PLA-0.4-10-210	0.4	10	210	5.0	12,700	OSB
3	PLA-0.4-10-210	0.4	10	210	5.0	18,000	
4	PLA-0.4-10-210	0.4	10	210	7.5	24,000	
5	PLA-0.4-10-210	0.4	10	210	10.0	4,200	
6	PLA-0.4-10-210	0.4	10	210	12.5	3,400

**Table 15:** Fatigue testing results for 3D-printed samples with a printing speed of 10 mm/s at 240 °C and a nozzle diameter of 0.4 mm.

No.	Material	Diameter	Speed	Temperature	Stress	Lifetime	Description
[-]	[-]	[mm]	[mm/s]	[˚C]	[MPa]	[cycle]	[-]
1	PLA-0.4-10-240	0.4	10	240	2.5	39,500	
2	PLA-0.4-10-240	0.4	10	240	5.0	10,000	
3	PLA-0.4-10-240	0.4	10	240	5.0	2,000	OSB
4	PLA-0.4-10-240	0.4	10	240	5.0	35,000	
5	PLA-0.4-10-240	0.4	10	240	7.5	4,000	
6	PLA-0.4-10-240	0.4	10	240	10.0	2,000

**Table 16:** Fatigue testing results for 3D-printed samples with a printing speed of 15 mm/s at 180 °C and a nozzle diameter of 0.4 mm.

No.	Material	Diameter	Speed	Temperature	Stress	Lifetime	Description
[-]	[-]	[mm]	[mm/s]	[˚C]	[MPa]	[cycle]	[-]
1	PLA-0.4-15-180	0.4	15	180	5.0	270,900	
2	PLA-0.4-15-180	0.4	15	180	7.5	27,400	
3	PLA-0.4-15-180	0.4	15	180	10.0	4,600	
4	PLA-0.4-15-180	0.4	15	180	10.0	13,000	
5	PLA-0.4-15-180	0.4	15	180	12.5	5,200	
6	PLA-0.4-15-180	0.4	15	180	15.0	2,300

**Table 17:** Fatigue testing results for 3D-printed samples with a printing speed of 15 mm/s at 210 °C and a nozzle diameter of 0.4 mm.

No.	Material	Diameter	Speed	Temperature	Stress	Lifetime	Description
[-]	[-]	[mm]	[mm/s]	[˚C]	[MPa]	[cycle]	[-]
1	PLA-0.4-15-210	0.4	15	210	5.0	48,700	
2	PLA-0.4-15-210	0.4	15	210	7.5	21,600	
3	PLA-0.4-15-210	0.4	15	210	10.0	5,300	
4	PLA-0.4-15-210	0.4	15	210	10.0	5,000	
5	PLA-0.4-15-210	0.4	15	210	12.5	7,000	
6	PLA-0.4-15-210	0.4	15	210	15.0	2,000

**Table 18:** Fatigue testing results for 3D-printed samples with a printing speed of 15 mm/s at 240 °C and a nozzle diameter of 0.4 mm.

No.	Material	Diameter	Speed	Temperature	Stress	Lifetime	Description
[-]	[-]	[mm]	[mm/s]	[˚C]	[MPa]	[cycle]	[-]
1	PLA-0.4-15-240	0.4	15	240	2.5	7,000	OSB
2	PLA-0.4-15-240	0.4	15	240	2.5	21,000	
3	PLA-0.4-15-240	0.4	15	240	5.0	24,000	
4	PLA-0.4-15-240	0.4	15	240	7.5	7,300	
5	PLA-0.4-15-240	0.4	15	240	10.0	4,300	
6	PLA-0.4-15-240	0.4	15	240	12.5	1,500

**Table 19:** Fatigue testing results for 3D-printed samples with a printing speed of 5 mm/s at 180 °C and a nozzle diameter of 0.6 mm.

No.	Material	Diameter	Speed	Temperature	Stress	Lifetime	Description
[-]	[-]	[mm]	[mm/s]	[˚C]	[MPa]	[cycle]	[-]
1	PLA-0.6-5-180	0.6	5	180	5.0	141,900	
2	PLA-0.6-5-180	0.6	5	180	7.5	21,000	
3	PLA-0.6-5-180	0.6	5	180	7.5	6,000	OSB
4	PLA-0.6-5-180	0.6	5	180	7.5	1,000	OSB
5	PLA-0.6-5-180	0.6	5	180	10.0	8,400	
6	PLA-0.6-5-180	0.6	5	180	12.5	5,500	
7	PLA-0.6-5-180	0.6	5	180	15.0	2,500

**Table 20:** Fatigue testing results for 3D-printed samples with a printing speed of 5 mm/s at 210 °C and a nozzle diameter of 0.6 mm.

No.	Material	Diameter	Speed	Temperature	Stress	Lifetime	Description
[-]	[-]	[mm]	[mm/s]	[˚C]	[MPa]	[cycle]	[-]
1	PLA-0.6-5-210	0.6	5	210	2.5	12,000	OSB
2	PLA-0.6-5-210	0.6	5	210	2.5	108,000	
3	PLA-0.6-5-210	0.6	5	210	5.0	27,000	
4	PLA-0.6-5-210	0.6	5	210	7.5	5,700	
5	PLA-0.6-5-210	0.6	5	210	10.0	1,200	
6	PLA-0.6-5-210	0.6	5	210	10.0	4,000

**Table 21:** Fatigue testing results for 3D-printed samples with a printing speed of 5 mm/s at 240 °C and a nozzle diameter of 0.6 mm.

No.	Material	Diameter	Speed	Temperature	Stress	Lifetime	Description
[-]	[-]	[mm]	[mm/s]	[˚C]	[MPa]	[cycle]	[-]
1	PLA-0.6-5-240	0.6	5	240	2.5	26,000	
2	PLA-0.6-5-240	0.6	5	240	5.0	11,500	
3	PLA-0.6-5-240	0.6	5	240	5.0	4,700	
4	PLA-0.6-5-240	0.6	5	240	7.5	1,600	OSB
5	PLA-0.6-5-240	0.6	5	240	7.5	2,800	
6	PLA-0.6-5-240	0.6	5	240	10.0	2,600

**Table 22:** Fatigue testing results for 3D-printed samples with a printing speed of 10 mm/s at 180 °C and a nozzle diameter of 0.6 mm.

No.	Material	Diameter	Speed	Temperature	Stress	Lifetime	Description
[-]	[-]	[mm]	[mm/s]	[˚C]	[MPa]	[cycle]	[-]
1	PLA-0.6-10-180	0.6	10	180	5.0	72,000	
2	PLA-0.6-10-180	0.6	10	180	7.5	16,500	
3	PLA-0.6-10-180	0.6	10	180	7.5	27,900	
4	PLA-0.6-10-180	0.6	10	180	10.0	17,400	
5	PLA-0.6-10-180	0.6	10	180	12.5	10,100	
6	PLA-0.6-10-180	0.6	10	180	15.0	6,000

**Table 23:** Fatigue testing results for 3D-printed samples with a printing speed of 10 mm/s at 210 °C and a nozzle diameter of 0.6 mm.

No.	Material	Diameter	Speed	Temperature	Stress	Lifetime	Description
[-]	[-]	[mm]	[mm/s]	[˚C]	[MPa]	[cycle]	[-]
1	PLA-0.6-10-210	0.6	10	210	5.0	57,000	
2	PLA-0.6-10-210	0.6	10	210	7.5	40,700	
3	PLA-0.6-10-210	0.6	10	210	10.0	6,000	
4	PLA-0.6-10-210	0.6	10	210	10.0	5,500	
5	PLA-0.6-10-210	0.6	10	210	12.5	4,200	
6	PLA-0.6-10-210	0.6	10	210	15.0	4,000

**Table 24:** Fatigue testing results for 3D-printed samples with a printing speed of 10 mm/s at 240 °C and a nozzle diameter of 0.6 mm.

No.	Material	Diameter	Speed	Temperature	Stress	Lifetime	Description
[-]	[-]	[mm]	[mm/s]	[˚C]	[MPa]	[cycle]	[-]
1	PLA-0.6-10-240	0.6	10	240	5.0	13,600	
2	PLA-0.6-10-240	0.6	10	240	5.0	34,100	
3	PLA-0.6-10-240	0.6	10	240	7.5	10,000	
4	PLA-0.6-10-240	0.6	10	240	10.0	3,900	
5	PLA-0.6-10-240	0.6	10	240	10.0	4,200	
6	PLA-0.6-10-240	0.6	10	240	12.5	2,000

**Table 25:** Fatigue testing results for 3D-printed samples with a printing speed of 15 mm/s at 180 °C and a nozzle diameter of 0.6 mm.

No.	Material	Diameter	Speed	Temperature	Stress	Lifetime	Description
[-]	[-]	[mm]	[mm/s]	[˚C]	[MPa]	[cycle]	[-]
1	PLA-0.6-15-180	0.6	15	180	5.0	39,600	
2	PLA-0.6-15-180	0.6	15	180	5.0	40,800	
3	PLA-0.6-15-180	0.6	15	180	7.5	10,000	
4	PLA-0.6-15-180	0.6	15	180	7.5	22,000	
5	PLA-0.6-15-180	0.6	15	180	10.0	10,000	
6	PLA-0.6-15-180	0.6	15	180	10.0	3,000	
7	PLA-0.6-15-180	0.6	15	180	12.5	2,400

**Table 26:** Fatigue testing results for 3D-printed samples with a printing speed of 15 mm/s at 210 °C and a nozzle diameter of 0.6 mm.

No.	Material	Diameter	Speed	Temperature	Stress	Lifetime	Description
[-]	[-]	[mm]	[mm/s]	[˚C]	[MPa]	[cycle]	[-]
1	PLA-0.6-15-210	0.6	15	210	5.0	27,400	
2	PLA-0.6-15-210	0.6	15	210	5.0	34,000	
3	PLA-0.6-15-210	0.6	15	210	7.5	16,800	
4	PLA-0.6-15-210	0.6	15	210	10.0	12,000	
5	PLA-0.6-15-210	0.6	15	210	12.5	6,700	
6	PLA-0.6-15-210	0.6	15	210	15.0	6,400	
7	PLA-0.6-15-210	0.6	15	210	17.5	3,200

**Table 27:** Fatigue testing results for 3D-printed samples with a printing speed of 15 mm/s at 240 °C and a nozzle diameter of 0.6 mm.

No.	Material	Diameter	Speed	Temperature	Stress	Lifetime	Description
[-]	[-]	[mm]	[mm/s]	[˚C]	[MPa]	[cycle]	[-]
1	PLA-0.6-15-240	0.6	15	240	5.0	18,000	
2	PLA-0.6-15-240	0.6	15	240	7.5	7,400	
3	PLA-0.6-15-240	0.6	15	240	7.5	5,000	OSB
4	PLA-0.6-15-240	0.6	15	240	10.0	2,700	OSB
5	PLA-0.6-15-240	0.6	15	240	10.0	5,200	
6	PLA-0.6-15-240	0.6	15	240	12.5	4,500	

## 2 Materials and Experimental Design

**Fig. 1:**
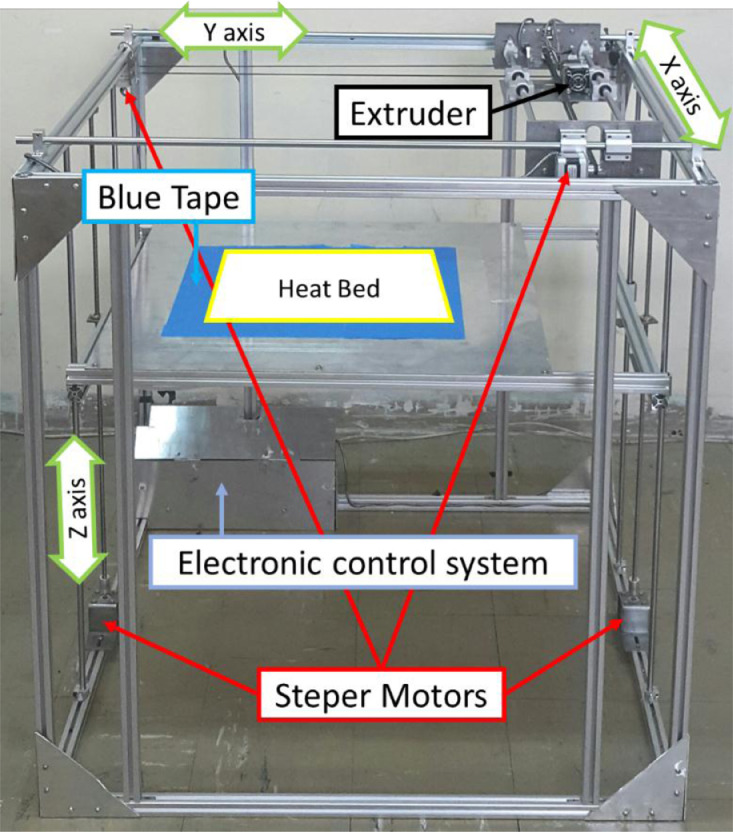
The FDM 3D-printer device.

**Fig. 2:**
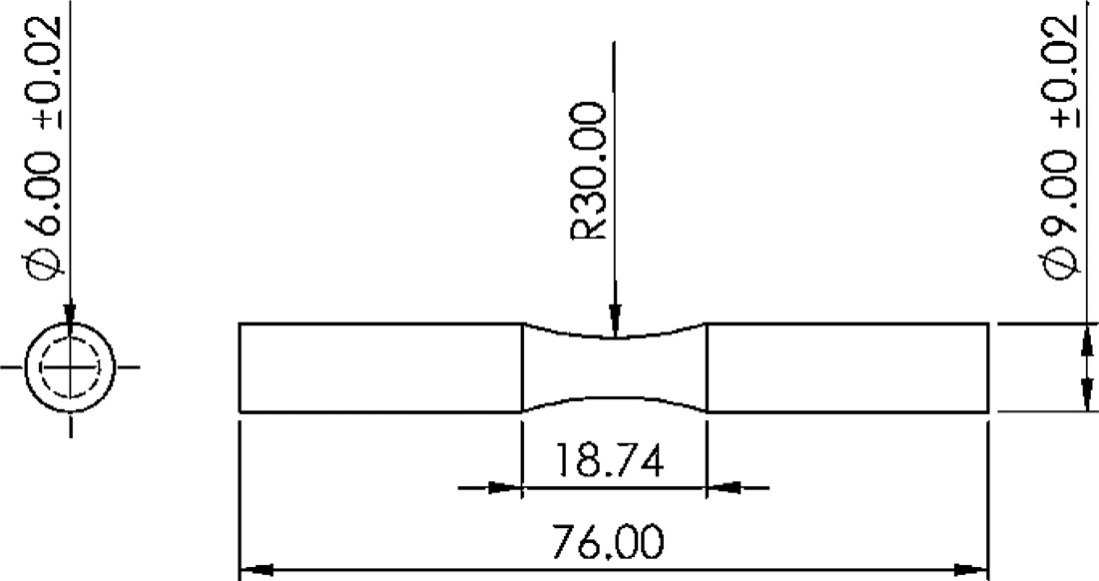
The geometry of standard samples for fatigue testing.

For the material, the transparent PLA filaments were used with 1.75 mm of diameter, made by YouSu Company. Then, the FDM 3D-printer (Fig. 1) was utilized to fabricate the fatigue testing samples. These specimens were cylindrical based on the ISO-1143 standard [1]. The geometry and the sample dimensions are presented in Fig. 2 in millimeters.

In this dataset, 3 parameters of FDM 3D-printing were considered as the nozzle diameter, the printing temperature, and the printing speed. Then, different standard samples were fabricated to consider all mentioned variables, which change in 3 levels. These descriptions could be seen in Table 28. It should be noted that other parameters of 3D-printing were constant for all specimens, as mentioned in Table 29. Such additive manufacturing parameters were also listed in the literature [2].

**Table 28:** Variable 3D-printing parameters.

Parameters	Level 1	Level 2	Level 3
Nozzle Diameter (mm)	0.2	0.4	0.6
3D-Printing Temperature (°C)	180	210	240
3D-Printing Speed (mm/s)	5	10	15

**Table 29:** Constant 3D-printing parameters.

Parameters	Values or Descriptions
Layer Thickness (mm)	0.2
Perimeter	2
Solid Layers	Top-1, Bottom-1
Fill Pattern	Rectangular
Travel Speed (mm/s)	30
Bed Temperature (°C)	30 (Room Temperature)
3D-Print Direction	Horizontal
Infill (%)	60

Due to the horizontal printing direction, the support has been used to provide the possibility of printing overhangs. In addition, a raft layer has been considered for proper adhesion of the initial layers to the print platform. Removing these materials affects the surface quality and consequently fatigue properties. Thus, post-processing was performed by polishing with sandpaper number of 120 to increase the surface quality for all samples.

**Fig. 3:**
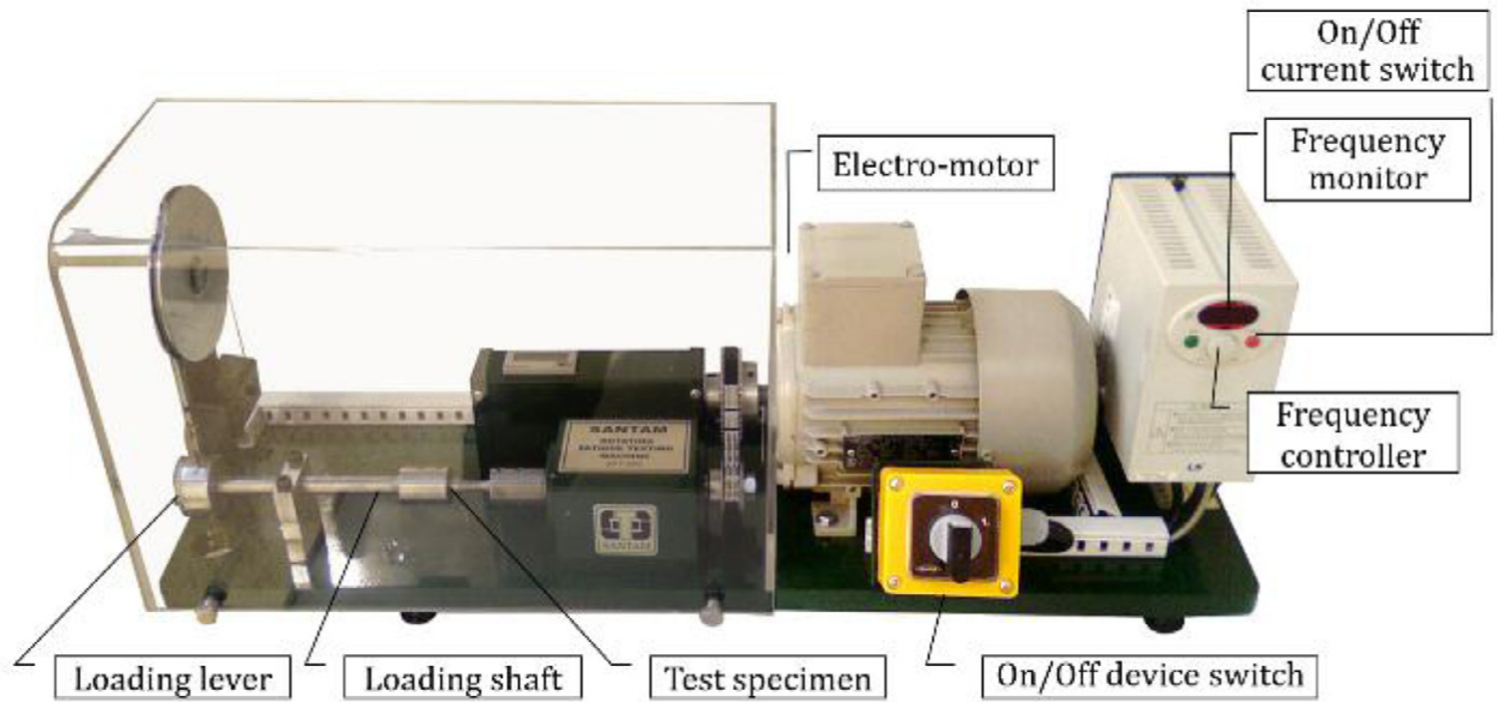
The device of SFT-600 Santam rotating-bending fatigue testing.

After fabricating PLA samples, fatigue testing was done under the load-controlled loading condition. For such an objective, the rotary bending fatigue test device (Santam Company, depicted in Fig. 3) was used under fully-reversed cyclic loadings. Under bending stress, the high-cycle fatigue (HCF) regime was considered for the material at room temperature. It should be mentioned that the frequency of cyclic loading was 100 Hz. As a note, although the temperature was not measured through testing, no high temperatures were sensed on samples after fatigue testing from the optical point of view, which also shows the uniaxiality of loading. Such similar data were presented for fatigue of polymers [3] or strengths of additive-manufactured materials [4] as the data-in-brief.

The use of fused deposition modeling can be developed in the future. The experimental data on the behavior and properties of this manufacturing process, such as residual stress and surface quality, are few and require more research.

## Ethics Statement

It is not applicable to this dataset.

## CRediT Author Statement

**Mohammad Azadi:** Conceptualization, Methodology, Investigation, Validation, Writing- Original draft preparation, Writing- Reviewing and Editing, Supervision. **Ali Dadashi:** Data curation, Software, Writing- Original draft preparation, Visualization, Investigation.

## Data Availability

The data that support the findings of this article are available at Azadi, Mohammad; Dadashi, Ali (2021), “HCF testing raw data on 3D-printed PLA polymers”, Mendeley Data, V1, DOI: 10.17632/gyxsn7wg6c.1.

## Declaration of Competing Interest

The authors declare that they have no known competing financial interests or personal relationships that could have appeared to influence the work reported in this paper.

## Appendix Supplementary materials

Image, application 1
